# Unleashing the power of visible‐near infrared spectroscopy: predicting Golden Delicious apple enzyme activity

**DOI:** 10.1002/jsfa.70782

**Published:** 2026-06-09

**Authors:** Tarahom Mesri Gundoshmian, Mahsa Sadat Razavi, Reza Akbari, Mohammad Tahmasebi, Irene Locatelli, Silvia Grassi

**Affiliations:** ^1^ Department of Biosystems Engineering University of Mohaghegh Ardabili Ardabil Iran; ^2^ Department of Food, Environmental, and Nutritional Sciences (DeFENS) University of Milan Milan Italy

**Keywords:** Golden Delicious apples, machine learning, metaheuristic algorithms, non‐destructive quality assessment, peroxidase, polyphenol oxidase, visible‐NIR spectroscopy

## Abstract

**BACKGROUND:**

Enzymatic browning is a significant reaction in fruits that affects their color, appearance, and quality. The quality of apples, as a perishable product, is mainly influenced by the activity of two browning‐related enzymes, polyphenol oxidase (PPO) and peroxidase (POD), during storage. Assessment of these enzymes using conventional methods is often destructive and time‐consuming, preventing rapid and non‐invasive monitoring of fruit quality. In this study, a visible‐near infrared (visible‐NIR) spectroscopy approach was developed to predict the enzymatic activity of PPO and POD in intact Golden Delicious apples, aiming to enable rapid, non‐destructive evaluation and to identify the most informative spectral regions for industrial applications.

**RESULTS:**

Both support vector regression (SVR) and decision tree (DT) algorithms achieved high performance when combined with non‐linear feature selection algorithms. The best performance, in terms of elapsed time and figure of merits, was achieved by combining particle swarm optimization (PSO) with SVR and DT. However, partial least squares (PLS) models outperformed both SVR‐PSO and DT‐PSO.

**CONCLUSIONS:**

This study is an advanced proof of concept of the use of visible‐NIR spectroscopy – combined with variable selection and machine learning algorithms – for predicting browning‐related enzyme activity in apples. The SVR and DT algorithms, coupled with metaheuristic strategies, reached lower performances than PLS, but the success of the variable selection strategy lays the groundwork for developing a miniaturized sensor for assessing apple quality during storage and controlling browning. © 2026 The Author(s). *Journal of the Science of Food and Agriculture* published by John Wiley & Sons Ltd on behalf of Society of Chemical Industry.

## INTRODUCTION

The consumption of fruits and vegetables has surged in recent years due to their abundant nutrient profile essential for human health.^1^ Among these, apples, renowned globally for their nutritional value, are rich in vitamins, minerals, and bioactive compounds.^2^ However, a significant challenge in the apple industry is the persistent gap between yield and quality. This disparity arises primarily from the limited adoption of advanced technologies, inadequate quality monitoring tools, and inefficient sorting systems.^3^ Modern consumers, beyond visual appeal and external quality, prioritize internal quality attributes such as texture, internal defects, and the content of beneficial compounds like vitamins.^4^

Two pivotal enzymes, polyphenol oxidase (PPO) and peroxidase (POD), significantly influence the quality of pome fruits (e.g., apples, pears, and quinces) and their postharvest properties. PPO is well‐known for its role in enzymatic browning,^5^ while POD contributes to undesirable changes in the flavor, texture, and color of processed fruits.^6^ Enzymatic browning, occurring during handling, storage, and processing, deteriorates quality, reduces market value, and adversely affects the color, flavor, and texture of fruits.^7^ Approximately 50% of fruits are discarded due to quality defects and browning, primarily catalyzed by PPO.^8^ Given their central role in enzymatic browning, PPO and POD are of paramount importance in the food industry. These enzymes not only alter the internal color of fruits but also affect their external appearance. PPO and POD catalyze the hydroxylation of monophenols to o‐diphenols and the oxidation of o‐diphenols to o‐quinones, respectively. Subsequently, o‐quinones react with each other to form melanins, the pigments responsible for the dark brown color.^9^ Enzymatic browning compromises the visual appeal of fruits and also negatively impacts their flavor, aroma, and nutritional value. Therefore, the food industry continually seeks methods to inactivate PPO and POD and prevent this undesirable process. A number of anti‐browning compounds have been studied to treat fresh‐cut fruits.^10^ However, its occurrence in intact fruits is faced by handling fruits with improved preharvest and postharvest storage control, which limit but do not eliminate bruising occurrence, and consequent quality decay.^11^ Traditional assessment methods, such as visual inspection, are insufficient for evaluating these internal characteristics, and destructive chemical analyses render the fruit unsuitable for consumption.^12^ Consequently, the implementation of non‐destructive, rapid, and online methods to monitor fruit quality throughout the supply chain is imperative. These methods should continuously assess product quality and ensure the delivery of high‐quality fruits to consumers.

Among these, visible‐near‐infrared (visible‐NIR) spectroscopy is a well‐established non‐destructive technology already applied to assess fruit and vegetable attributes,^13^, ^14^, ^15^ However, challenges remain in processing and analyzing the complex spectral data, especially when simplified instruments and software are envisioned. To address these challenges, various variable selection methods have been developed, including synergy interval,^16^ weighted iterative sampling,^17^ ant colony optimization,^18^ uninformative variable elimination,^19^ and successive projection algorithm.^20^ These methods simplify the model and reduce data dimensionality, improving modeling accuracy and stability, and enabling the practical application of NIR spectroscopy by miniaturized instrumentation. Furthermore, non‐linear optimization algorithms have demonstrated their effectiveness in improving variable selection by adaptively discovering relevant features.^21^ In particular, the combination of metaheuristic strategies with machine learning models has recently shown promising results for the non‐destructive estimation of PPO and POD activity in horticultural products such as bell peppers^22^ and melon leaves,^23^ confirming the potential of spectral approaches for rapid enzyme monitoring across different food matrices.

Given the paucity of research on optimizing spectral models using non‐linear algorithms to predict the activity of PPO and POD enzymes in apples, this study investigates the application of visible‐NIR spectroscopy to estimate the activity of PPO and POD in Golden Delicious apple with the final aim of selecting the most informative wavelengths to be implemented in a multichannel point instrument.

## MATERIALS AND METHODS

### Sample preparation

Apples were manually harvested from an orchard located in Mianeh County, Ardabil Province, northwestern Iran. One hundred undamaged, microbiologically sound apples of uniform size were selected and stored in plastic bags at 4 ± 0.5 °C until analysis. Two sampling campaigns were organized: on the first sampling day 80 apples were used for model calibration and validation (test set 1), in the second sampling day 20 apples were used for model validation purposes (test set 2). Prior to analysis, samples were equilibrated at room temperature for 24 h. After washing, samples were allowed to air‐dry at room temperature. Subsequently, juice samples containing pulp were prepared at 23 ± 0.5 °C and used for destructive measurements as described later.

### Visible‐NIR spectroscopy

Visible‐NIR spectra of the apples were acquired using a PS‐100 visible‐NIR spectrophotometer manufactured by Apogee Instruments (Logan, UT, USA). Equipped with a 2048‐pixel charge coupled device (CCD) detector and a spectral resolution of 1 nm, the instrument employed a halogen‐tungsten light source operating within a wavelength range 350–1150 nm.

**Figure 1:**
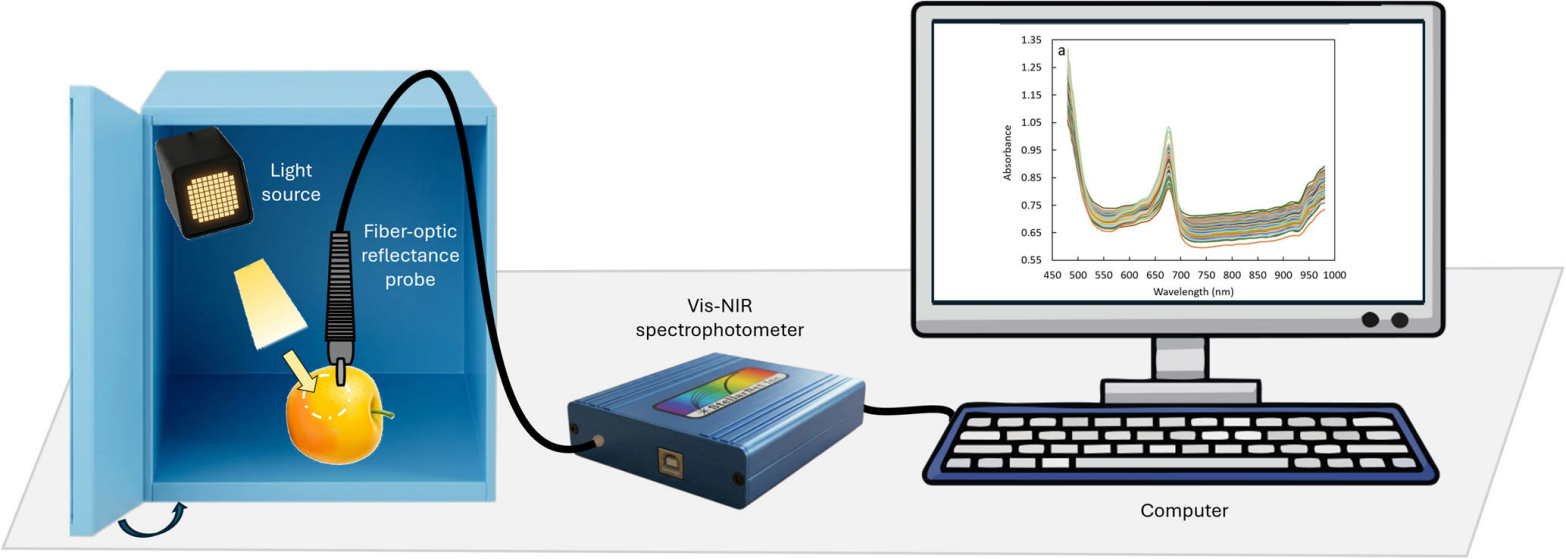
Schematic representation of the visible‐NIR experimental setup used for non‐destructive spectral acquisition on intact Golden Delicious apples. The apple is positioned inside a light‐isolated chamber. Spectra were acquired using a fiber‐optic probe in direct contact with the fruit surface, connected to the spectrophotometer, and the data acquisition system.

To perform measurements (Fig. 1), whole, intact apples were placed inside a light‐isolated chamber in order to minimize interference from external light sources. Spectra were acquired using a fiber optic probe positioned in direct contact with the fruit surface at two orthogonal points on each sample. Spectra were acquired in the range 480–980 nm. To calibrate the instrument, black (Teflon) and white reference spectra were recorded under dark and light conditions using standard calibration plates.

A total of 200 spectra were collected (two points per sample for 100 samples), ensuring uniform coverage of the fruit surface and minimizing variability due to fruit orientation.

### Polyphenol oxidase (PPO) and peroxidase (POD) enzyme activity

Enzyme extracts were prepared from fruit tissue using 0.4 mol L^−1^ sodium phosphate buffer (pH 6.5). The enzyme extraction solution was then prepared by adding 40 g kg^−1^ polyvinylpolypyrrolidone (PVPP) and 1 mL L^−1^ Triton X‐100 to 0.2 mol L^−1^ sodium phosphate buffer (pH 6.5). Finally, the fruit tissues were homogenized, and 10 g of extract was mixed with 20 g of enzyme extraction solution, followed by centrifugation at 7500 × *g* for 10 min at 4 °C (LISA, Château‐Gontier, France). The supernatant was collected for enzyme activity measurements.

For each apple, two independent extractions were performed, corresponding to the two orthogonal portions of the samples used in the visible‐NIR analysis. For each extraction, five replicates were carried out.

The standard error of laboratory (SEL) was estimated from replicate measurements (*n* = 5) as the standard deviation divided by the square root of the number of replicates (SD/√*n*) and used as an indicator of the analytical variability of the reference measurements.^24^

To determine POD activity, 500 μL of supernatant was mixed with 1 mL of 0.05 mol L^−1^ sodium phosphate buffer (pH 6.5). The reaction was initiated by adding 1 mL of 0.05 mol L^−1^ sodium phosphate buffer containing 1 g kg^−1^ *p*‐phenylenediamine and 500 μL of 15 mL L^−1^ hydrogen peroxide. Absorbance at 485 nm was measured using a spectrophotometer (NanoDrop One C; Thermo Scientific, Waltham, MA, USA) at 25 °C in kinetic mode for 10 min. POD activity was expressed as micrograms of oxidized *p*‐phenylenediamine per gram of fresh weight per minute (μg g^−1^ min^−1^), calculated from the change in absorbance using the molar extinction coefficient.^25^

For PPO enzyme activity, 75 μL of supernatant was combined with 3 mL of 0.05 mol L^−1^ sodium phosphate buffer containing 0.07 mol L^−1^ catechol. For the control treatment, a similar procedure was followed, but distilled water was used instead of the enzyme extraction solution. Kinetic absorbance at 420 nm was measured using a spectrophotometer (NanoDrop One C; Thermo Scientific) at room temperature for 10 min. Enzyme activity was finally expressed as the change in absorbance per minute per gram of sample (absorbance min^−1^ g^−1^).^25^

Chemicals used in the chemical analyses included monosodium dihydrogen phosphate (NaH_2_PO_4_), disodium hydrogen phosphate (Na_2_HPO_4_), PVPP, Triton X‐100, catechol, *p*‐phenylenediamine, and hydrogen peroxide, all obtained from Merck (Darmstadt, Germany).

### Spectral data analysis and variable selection using metaheuristic algorithms

Principal component analysis (PCA) was employed to explore the spectral data after column mean centering. Subsequently, outliers, attributed to experimental and measurement errors, were identified and removed from the spectral data using PCA in conjunction with Hotelling's *T* ^2^ and *Q*‐residual diagnostics. Given the negligible noise level in the spectra and the minimal impact of preprocessing, row preprocessing steps were omitted.

Partial least squares regression (PLSR) models were developed as a benchmark for quantitative prediction, due to their widely used coupling with NIR spectroscopy.^26^ Its primary objective is to identify a limited number of linear combinations that can maximally explain the variance in both the predictor (X) and response (Y) datasets, where X represents the spectra and Y represents the quality attributes of the apples.

The PLSR models were built, after removing the outliers, considering a calibration dataset consisting of around 70% of apples used on day 1 (97 spectra), cross‐validated with the same calibration set. Finally, the model was externally validated by two independent datasets: test set 1, around 30% of samples obtained on day 1 (test set 1, 41 spectra); and test set 2, spectra collected from apples acquired on day 2 (test set 2, 36 spectra).

The regression model performance was evaluated using a comprehensive set of figures of merits (FoM), including root mean square error (RMSE) and coefficient of determination (R^2^) in calibration, cross‐validation and prediction, ratio of performance to deviation (RPD), and bias.^27^

Different variable selection methods, capable of reducing the model to a smaller subset of wavelengths, were studied to simplify models, decrease computational time, and enhance the stability of parameter predictions.^28^ The entire dataset was first used to select effective wavelengths (EWs). Consequentially the dataset was split – as for the partial least squares (PLS) modeling – into a calibration dataset consisting of around 56% of the total data (97 samples), and in two independent datasets of around 23% of the total data (41 samples) and 21% of spectra (36 samples).

To develop predictive models and identify relevant wavelengths, the decision tree (DT) and support vector regression (SVR) models were individually combined with various feature selection methods, including different metaheuristic strategies, such as genetic algorithm (GA), particle swarm optimization (PSO), ant colony optimization (ACO), and imperialist competitive algorithm (ICA).

The most promising solution was selected considering FoM, execution speed, and minimal number of relevant variables.

DT models were developed as machine learning approach which can handle non‐linear relationships. Their structure is constituted of a root node, branches, internal nodes, and leaf nodes.^29^ DT predicts a continuous outcome by recursively splitting the data into regions based on input variables and assigning each region the average target value of its training samples. DT recursively splitting results in a computationally expensive and memory‐intensive strategy when dealing with large datasets.^29^

SVR algorithms are widely used supervised learning algorithms rooted in statistical learning theory, and they are indicated for handling high‐dimensional input spaces to find non‐linear global solutions in noisy data.^30^

Data processing and modeling were conducted using Matlab2023a (MathWorks Inc., Natick, Massachusetts, USA) environment and the PLS toolbox (Eigenvector Research, Inc., Manson, Washington, USA) for PLSR, and FeatureSelect toolbox^31^ for DT and SVR model combined with metaheuristic strategies.

## RESULTS AND DISCUSSION

### Destructive chemical analysis results for PPO and POD activities

Table 1 presents the results of the chemical analyses for PPO and POD enzyme activities. In addition to enzyme activities, the table reports the main physio‐chemical parameters of the samples (pH, titrable acidity and soluble solid content), together with the SEL, estimated from replicate measurements, to describe the analytical variability.

**Table 1:** Descriptive statistics (minimum, maximum, mean, standard deviation) of enzyme activities and physio‐chemical parameters measured in Golden Delicious apples (sample = 100) using standard analytical methods

	Minimum	Maximum	Mean	SD	SEL
POD (μg g^−1^ min^−1^)	6.513	8.468	7.462	0.646	0.03
PPO (absorbance min^−1^ g^−1^)	5.045	6.402	5.806	0.414	0.01
pH	3.21	3.84	3.84	0.40	0.02
TA (g kg^−1^)	1	1.19	1.09	0.05	0.01
SSC (°Brix)	8.5	16.20	12.47	2.12	0.07

*Note:* Standard deviation (SD), standard error of laboratory (SEL), peroxidase (POD) and polyphenol oxidase (PPO) enzyme activity, titratable acidity (TA) and soluble solids content (SSC).

As observed, the enzyme activity showed a relatively narrow range, with POD varying between 6.51 and 8.47 μg g^−1^ min^−1^ and PPO between 5.05 and 6.40 absorbance min^−1^ g^−1^. This limited variability can be attributed to the simultaneous harvest, and similar growth rates in terms of appearance and physiology, which are the expected ones. However, for improved spectral discrimination and model robustness, a wider variability in the reference values is generally desirable.^32^ The low SEL values further confirm the good repeatability of the reference methods and the reliability of the analytical measurements.

Previous studies on various apple cultivars and other products have reported a wide range of average values for enzyme activities, which are strongly influenced by cultivar, harvesting practices, and postharvest storage conditions. However, a direct quantitative comparison between literature data and the results obtained in the present study is limited due to differences in the units of measurement and analytical expressions used for enzyme activity. In the present study, PPO and POD activities are consistently reported as absorbance min^−1^ g^−1^ fresh weight and μg g^−1^ min^−1^, respectively, to ensure internal comparability and methodological consistency. In contrast, Wang *et al*.^33^ reported PPO activity as 2.8 × 10^3^ U kg^−1^ in apples at the ripening stage, and Eid *et al*.^34^ expressed PPO activity as 6.076 ± 0.092 μmol min mL^−1^ under similar extraction conditions. Serra *et al*.^35^ also reported POD and PPO activities using different units (units per gram of fresh weight) across 15 apple cultivars. Therefore, due to the lack of standardized units and assay conditions, these values are mainly comparable in a qualitative manner, highlighting general trends rather than allowing for a strict numerical comparison.

### Spectra exploration

**Figure 2:**
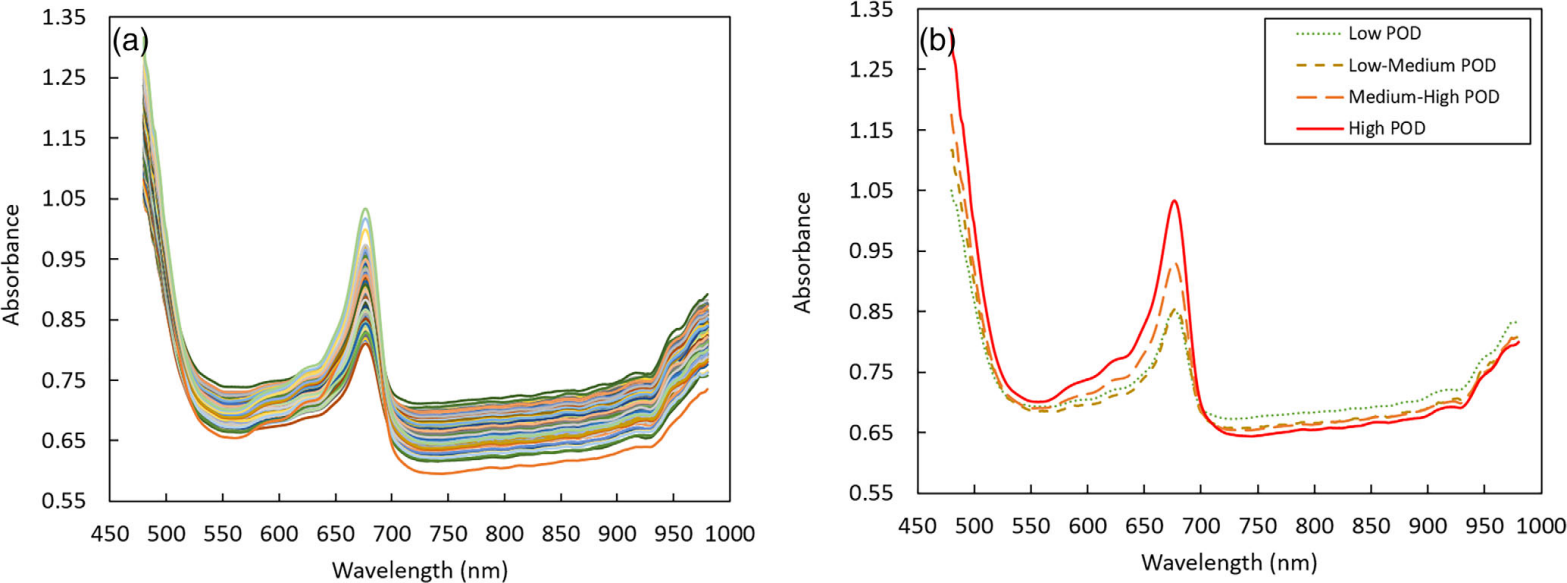
Visible‐NIR spectra (480–980 nm) of intact Golden Delicious apples. (a) Full set of calibration spectra, (b) four representative spectra selected across the range of POD activity: low (6.54 μg g^−1^ min^−1^), low‐medium (6.85 μg g^−1^ min^−1^), medium‐high (7.5 μg g^−1^ min^−1^), high (8.38 μg g^−1^ min^−1^).

Original visible‐NIR spectra collected for apples used in the calibration phase (Fig. 2(a)) are characterized by a main absorption peak at 680 nm, likely related to the third‐order doubling of the O–H bond (4*ν*), and the fourth‐order doubling of the C–H bond stretching vibration (5*ν*). This peak could be associated with fruit chlorophyll content.^36^ In addition, small absorption features are visible between 600 and 650 nm, and their intensity appears to increase with higher POD and PPO activity (Fig. 2(b)), suggesting a link to enzymatic browning and phenolic content.

**Figure 3:**
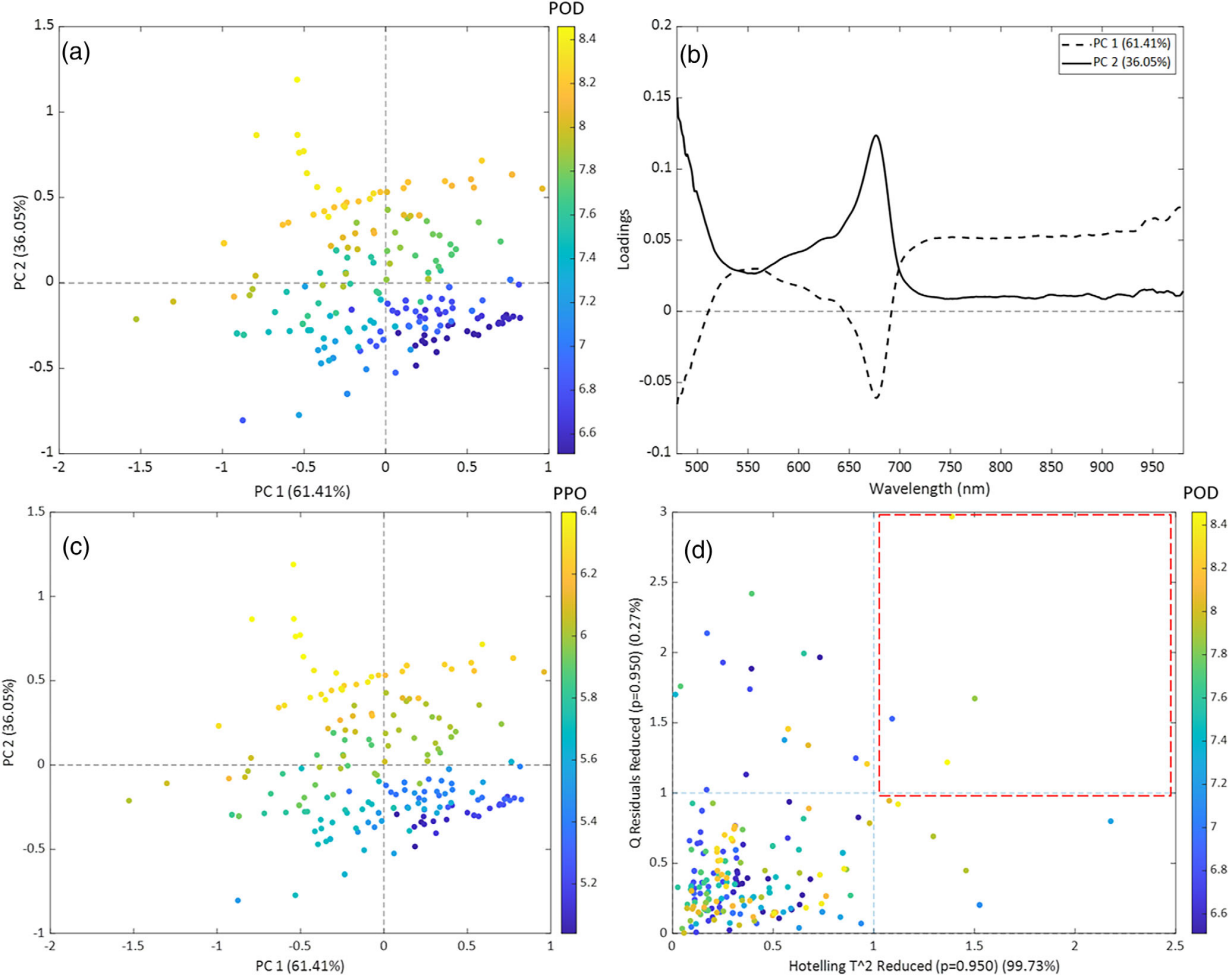
PCA of the visible‐NIR spectral data after column mean centering. (a) PC1 *versus* PC2 scores plot with samples colored according to POD activity, (b) PC1 and PC2 loadings plots, (c) PC1 *versus* PC2 scores plot with samples colored according to PPO activity, (d) Hotelling's *T* ^2^ *versus Q*‐residual plot, with 95% confidence limits (red hatched box) highlighting the outliers.

To highlight difference among samples and identify the spectral variables responsible for their differentiation, the spectra were investigated by PCA. Principal components one and two (PC1 and PC2), as illustrated in Fig. 3(a),(c), explained 61.4% and 36.1% of the total variance in the apple samples, respectively, together accounting for the majority of the spectral information. The score plots for POD (Fig. 3(a)) and PPO (Fig. 3(c)) show a nearly identical sample distribution, suggesting a strong correlation between the two enzyme activities.

Samples with low POD and PPO activity are characterized by positive PC1 and negative PC2 scores (IV quadrant), while samples with increasing enzyme activity show progressively higher PC2 scores, with the highest activity levels clustering in the II quadrant. This distribution suggests a consistent spectral gradient associated with enzyme activity across the sample set.

The loadings plots (Fig. 3(b)) reveal the spectral variables driving this separation. PC1 and PC2 loadings plots are dominated by the main spectral feature at 680 nm, associated with chlorophyll content. Just before this peak, in the region between 600 and 650 nm, the loadings show a slight change in slope which could be probably linked to variations driven by PPO and POD activity. Although smaller than the chlorophyll‐related feature, this spectral region contributes to distinguishing samples with different enzyme levels.

Furthermore, PC2 loadings are affected by slope changes from 720 to 980 nm, as already observed by Li *et al*.,^37^ likely reflects contribution from water content and C–H bond overtones.

Outliers' detection was performed using the Hotelling's *T* ^2^ statistic plotted against *Q‐*residuals (Fig. 3(d)). The *T* ^2^ distribution measures each sample's distance within the PCA model space, while *Q*‐residuals quantify the variance not captured by the retained components. The large majority of samples cluster in the lower‐left region with low values for both statistics, confirming a good fit of the PCA model to the spectral data. The data points exceeding the confidence limits (*P* = 0.95) of Hotelling's *T* ^2^ or *Q*‐residuals, that is, 26 spectra, were identified as outliers and excluded from subsequent analyses.

### Partial least squares regression (PLSR)

PLS algorithm was employed to construct regression models and consider the Golden standard. The optimal number of latent variables (LVs) was determined by minimizing the RMSEs in calibration and cross‐validation through the scree plot. The performance of the models was evaluated based on correlation coefficients and RMSEs across all phases. As detailed in the Materials and Methods section, two independent external test sets were used: test set 1 (~30% of apples from day 1) and test set 2 (apples from the second sampling day). The FoM for all the phases are presented in Table 2.

**Table 2:** Figure of merits of the partial least squares regression (PLSR) models for peroxidase (POD) and polyphenol oxidase (PPO) enzyme activity prediction in intact Golden Delicious apples, evaluated on the calibration set, cross‐validation and two external prediction sets

Parameter	LVs	Calibration		Cross‐validation		Prediction			
		*R* ^*2*^_cal_	RMSE	*R* ^*2*^_cv_	RMSE	*R* ^*2*^_pred_	RMSE	*R* ^*2*^_pred_	RMSE
POD (μg g^−1^ min^−1^)	4	0.98	0.081	0.98	0.090	0.98	0.079	0.98	0.081
PPO (absorbance min^−1^ g^−1^)	4	0.99	0.044	0.98	0.049	0.99	0.040	0.98	0.039

*Note:* Coefficient of determination (*R* ^2^), root mean square error (RMSE), latent variables (LVs).

The models demonstrated high stability and performance ability in calibration, cross‐validation and in both external tests set prediction, having *R* ^2^ of 0.98–0.99 and RMSE below 0.082 μg g^−1^ min^−1^ for POD and below 0.044 absorbance min^−1^ g^−1^ for PPO.

The RMSE of both external tests set prediction for POD and PPO were compared with the SEL of the reference methods reported in Table 1. For POD, the root mean square error of prediction (RMSEP) was approximately 2.7 times higher than the corresponding SEL (0.03 μg g^−1^ min^−1^), while for PPO the RMSEP was approximately 3.9 times higher than the SEL (0.01 absorbance min^−1^ g^−1^). These results indicate that the predictive models achieved a good level of accuracy, considering the intrinsic uncertainty of the reference methods, and confirm the reliability of the proposed approach for enzyme activity estimation.

Previous studies on enzyme activity in various products using PLSR have reported varying *R* ^2^ and RMSE values. Li and Hu^38^ employed PLSR to measure POD activity in potato leaf samples using hyperspectral data, obtaining quite high RMSEP (0.407 g kg^−1^) with a variation range 0.44–5.37 g kg^−1^. Similarly, Gaston *et al*.^39^ used PLSR to predict PPO activity in button mushrooms from hyperspectral imaging data, reporting the best RPD of 1.22, which cannot be considered successful. PLSR has also been used in combination with other methods to estimate PPO activity in tomatoes^37^ reaching 3.32 U mL^−1^ of RMSEP.

### Feature selection by metaheuristic algorithms combined with DT and SVR

To identify the most influential wavelengths for predicting enzyme activity, a combination of support metaheuristic algorithms was employed in combination with SVR and DT regressors. The different algorithms applied to the entire dataset yielded varying numbers of EWs (Table 3). Selecting fewer EWs reduces model complexity, enhances interpretability, and mitigates noise and interference. Furthermore, shorter computational times for spectral data processing and analysis are crucial for real‐time and online applications, especially in industrial and commercial settings. This is because processing data from thousands or millions of products at a large‐scale place significantly demands systems. Consequently, reduced processing time consequently decreases computational load, reduces equipment wear and tear, extends equipment lifespan, and minimizes costs.^40^

**Table 3:** Effective wavelengths (EWs) selected and computational time for each combination of metaheuristic algorithm (GA, PSO, ACO and ICA) and regressor (SVR and DT) for peroxidase (POD) and polyphenol oxidase (PPO) activity prediction

Enzyme	Regressor	MA	Elapsed time (s) for one run	Number of EWs	EWs (nm)
POD	SVR	GA	3.94	5	807, 732, 957, 480, 520
	SVR	PSO	4.00	5	671, 950, 819, 738, 643
	SVR	ACO	87.09	8	666, 498, 681, 807, 678, 497, 801, 490
	SVR	ICA	3.61	5	573, 766, 717, 629, 979
PPO	SVR	GA	4.41	5	893, 934, 615, 480, 694
	SVR	PSO	3.67	4	748, 952, 905, 600
	SVR	ACO	99.8	6	980, 639, 480, 851, 887, 725
	SVR	ICA	3.99	6	911, 735, 613, 798, 647, 582
POD	DT	GA	12.28	8	639, 740, 480, 980, 523, 771, 827, 912
	DT	PSO	7.19	10	837, 912, 803, 651, 922, 711, 656, 926, 524, 980
	DT	ACO	101.64	10	664, 851, 912, 557, 980, 547, 548, 575, 759, 616
	DT	ICA	7.32	9	732, 945, 980, 659, 969, 672, 618, 848, 892
PPO	DT	GA	13.91	7	952, 938, 930, 980, 747, 801, 967
	DT	PSO	7.21	8	980, 712, 538, 674, 816, 557, 959, 707
	DT	ACO	87.59	9	972, 716, 590, 527, 919, 616, 658, 581, 480
	DT	ICA	6.34	6	679, 707, 960, 584, 801, 554

*Note:* Metaheuristic algorithm (MA), support vector regression (SVR), decision tree (DT), genetic algorithm (GA), particle swarm optimization (PSO), ant colony optimization (ACO), imperialist competitive algorithm (ICA).

Table 3 reports the number of EWs (in nanometers) selected by each algorithm‐regressor combination, along with the elapsed time for one run. For SVR models, GA, PSO, and ICA consistently selected 5–6 wavelengths and achieved the shortest computation times (~4 s), whereas ACO required more EWs (6–8) and longer run times (~88–100 s).

For DT models, the number of EWs was generally higher (6–10), and run times were longer (~6–14 s), reflecting the greater complexity of tree‐based models. Within DT, ICA and PSO produced relatively shorter elapsed times compared to GA and ACO.

The performance of the selected EWs was evaluated based on two statistical criteria: the mean convergence correlation (ACC) and the mean convergence error (ACE).

Convergence analysis revealed clear differences among the metaheuristic algorithms: GA consistently exhibited the poorest performance, with the highest ACE values and the lowest ACC values for both enzymes (POD and PPO) and regressors (SVR or DT). In contrast, PSO demonstrated the most favorable convergence behavior, with a progressive decrease in ACE and consistent increase in ACC across iterations, outperforming GA, ACO, and ICA in most cases. ACO and ICA generally exhibited intermediate performance. Convergence trends for POD and PPO are reported in Supporting Information, Figs S1 and S2.

Overall, these results indicate that PSO provides the most robust and stable convergence behavior for enzyme activity prediction under the investigated conditions.

### Regression modeling using PSO‐selected wavelengths

Based on the convergence analysis, PSO was selected as the most effective metaheuristic algorithm and subsequently employed for the final regression modeling in combination with SVR and DT. The predictive performance of the PSO‐based model is reported in Table 4, following the same dataset split adopted for PLSR.

**Table 4:** Figures of merit of particle swarm optimization (PSO)‐based support vector regression (SVR) and decision tree (DT) models for peroxidase (POD) and polyphenol oxidase (PPO) enzyme activity prediction using PSO‐selected effective wavelengths

Enzyme	Method	Training						External validation (1)						External validation (2)					
		*R* ^2^	RMSE	Slope	Bias	RMSEP	RPD	*R* ^2^	RMSE	Slope	Bias	RMSEP	RPD	*R* ^2^	RMSE	Slope	Bias	RMSEP	RPD
POD (μg g^−1^ min^−1^)	SVR	0.99	0.077	0.945	−0.01	0.078	8.241	0.97	0.119	0.924	−0.004	0.119	5.418	0.87	0.196	0.98	−0.081	0.242	2.673
	DT	0.99	0.048	0.971	−0.016	0.055	11.739	0.92	0.244	1.065	−0.085	0.285	2.27	0.95	0.331	1.258	−0.19	0.467	1.385
PPO (absorbance min^−1^ g^−1^)	SVR	0.99	0.035	1.027	0.014	0.042	9.961	0.96	0.11	1.312	0.019	0.116	3.581	0.93	0.171	1.258	0.006	0.171	2.414
	DT	0.98	0.052	0.953	−0.001	0.052	7.927	0.97	0.078	0.868	−0.027	0.091	4.537	0.93	0.116	0.938	0.018	0.12	3.446

*Note:* Coefficient of determination (*R* ^2^), root mean square error (RMSE), root mean square error of prediction (RMSEP), ratio of performance to deviation (RPD). Results are reported for training and two external validation sets.

The good predictive accuracy achieved by both SVR‐PSO and DT‐PSO models confirms that an appropriate selection of EWs plays a crucial role in enhancing model performance while reducing spectral redundancy. In the external validation sets, *R* ^2^ values remained high for both POD and PPO, ranged approximately from 0.87 to 0.97, indicating that the models capture most of the variability in enzyme activity. The RMSE ranged from 0.12 to 0.33 for POD and from 0.08 to 0.17 for PPO.

The bias values were generally low, indicating minimal systematic deviation between predicted and observed enzyme activities, while the slopes of the regression lines were close to 1, confirming good linearity in both the calibration and validation phases. The RPD values further support the reliability of the models: in most cases, RPD values were higher than 2, indicating good predictive ability, although slightly lower values observed in some DT external validations suggest reduced robustness and potential overfitting.

However, the regression performance of both SVR and DT models resulted slightly lower than that obtained with the PLS models (Table 2). In particular, the stability of the FoMs along the developing phases (i.e., calibration and prediction) was reduced in SVR and DT models, indicating a higher tendency to overfit the data. This is also reflected in the prediction errors: the RMSEP values obtained with PLS models (0.081 μg g^−1^ min^−1^ for POD and 0.039 absorbance min^−1^ g^−1^ for PPO) were consistently lower than those obtained with SVR and DT models (Table 4).

When compared with the SEL (Table 1), the PLS model showed closer agreement with the analytical variability, whereas SVR and DT models exhibit larger deviations. This indicates that the PLS model provides a more accurate and reliable prediction of enzyme activity under the conditions examined, whereas PSO‐based models, despite reducing the number of variables and computational complexity, result in greater uncertainty in the prediction.

This is partially in contrast with previous studies demonstrating the effectiveness of combining metaheuristic or feature selection methods with regression algorithms for enzyme activity estimation. For instance, Li and Hu^38^ combined the SVR algorithm with the successive projection algorithm (SPA) to predict POD enzyme activity in potato leaves, reporting *R*_p_ and RMSEP values of 0.009 and 0.24 g kg^−1^, respectively, gaining predicting performances over the PLSR model. Mollazade^41^ applied competitive adaptive reweighted sampling (CARS) to hyperspectral data to assess browning in cultivated mushrooms, identifying 56 EWs from the full spectral range and subsequently reducing them to eight wavelengths for system simplification. Similarly, Yang *et al*.^42^ combined the SVR algorithm with recursive feature elimination (RFE) and highlighted the importance of selecting an optimal subset of wavelengths to maximize calibration and prediction performance for PPO estimation.

With specific reference to apple analysis by visible‐NIR spectroscopy, most investigations have focused on the wavelength range between 601 and 800 nm, particularly within 651–700 nm, for the estimation of quality‐related parameters.^43^ In sliced apples, 677 nm has been identified as an optimal wavelength for PPO prediction.^44^ Additionally, for button mushrooms, the 470–640 nm range has been reported to be relevant for PPO estimation.^41^ These findings suggest that wavelengths located in these spectral regions are strongly associated with biochemical changes related to enzyme activity.

In any case, the PSO‐based models using a reduced set of wavelengths could provide good predictive accuracy, demonstrating the advantage of combining feature selection with regression to reduce model complexity and maintain high performance.

## CONCLUSIONS

In this study, visible‐NIR spectroscopy was employed to estimate the activities of two enzymes, PPO and POD, in Golden Delicious apple fruit tissue. Subsequently, the enzymatic content of the fruits was measured using destructive reference methods. The data exploration by PCA suggested a distribution trend related to difference in enzymatic activity present in the visible regions of 600–650 nm and 720 to 980 nm, with the main influence of the band at 680 nm. Thus, PLSR models were developed as benchmark regression approach in the field of vis‐NIR spectroscopy. PPO prediction reached a *R* ^2^ of 0.98 and RMSEP of 0.039 absorbance min^−1^ g^−1^, considering a variation range from 5.05 to 6.4. POD prediction performed equally with an RMSEP of 0.081 μg g^−1^ min^−1^ considering a variation range from 6.5 to 8.5.

A tentative model optimization in terms of error, computational time, and variable (wavelength) reduction was conducted by combining SVR and DT algorithms with GA, PSO, ACO, and ICA.

Based on the lower number of EWs, minimal execution time, and maximum average convergence correlation (ACC close to 1 and ACE close to 0), PSO algorithm was selected for feature extraction. Both SVR‐PSO and DT‐PSO provided high trade‐off between efficiency and predictive accuracy. However, the PLS modeling resulted in highest performance, especially in model stability along testing phases.

The results demonstrate that combining visible‐NIR spectroscopy with PSO‐based feature selection and regression modeling enables computationally efficient prediction of enzymatic activity in apple tissue. Even if PLS modeling demonstrated a higher performance, the proposed approach could be implemented for rapid, non‐destructive quality assessment in industrial and commercial apple processing, focusing on the most informative wavelengths identified for each enzyme.

## CONFLICT OF INTEREST

The authors declare that they have no known competing financial interests or personal relationships that could have appeared to influence the work reported in this article.

## AUTHOR CONTRIBUTIONS

T Mesri Gundoshmian: writing – reviewing and editing, supervision, investigation. Mahsa S Razavi: conceptualization, methodology, software, validation, writing – original draft preparation, visualization. Reza Akbari: writing – reviewing and editing. Mohammad Tahmasebi: conceptualization, methodology, software, validation, writing – reviewing and editing, visualization, data curation. Irene Locatelli: writing – reviewing and editing, data curation, visualization. Silvia Grassi: writing – reviewing and editing data curation, validation.

## Supporting information


**Figure S1.** Convergence analysis of metaheuristic algorithms (GA, PSO, ACO, ICA) for POD activity prediction, combined with SVR (a, b) and DT (c, d) regressors. Panels (a) and (c) show the evolution of the mean convergence error (ACE, i.e., average RMSE) across iterations; panels (b) and (d) show the mean convergence correlation (ACC) across iterations.
**Figure S2**. Convergence analysis of metaheuristic algorithms (GA, PSO, ACO, ICA) for PPO activity prediction, combined with SVR (a, b) and DT (c, d) regressors. Panels (a) and (c) show the evolution of the mean convergence error (ACE) across iterations; panels (b) and (d) show the mean convergence correlation (ACC) across iterations.

## Data Availability

The data that support the findings of this study are available from the corresponding author upon reasonable request.
