# Supplementary material for: Unleashing the power of visible‐near infrared spectroscopy: predicting Golden Delicious apple enzyme activity
Source: J Sci Food Agric. 2026 Jun 9;106(12):7503–11. doi: 10.1002/jsfa.70782 (PMC13442799; doi:10.1002/jsfa.70782)
Supplement: Supplementary file 1 [file JSFA-106-7503-s001.docx]

**Figure S1.** Diagrams produced based on the POD activity using algorisms combined with (a,b) SVR and (c,d) DT for the “Golden Delicious” apples These diagrams compare the performance of algorithms in terms of RMSE variations and average correlation.

**Figure S2.** Diagrams produced based on the PPO activity using algorisms combined with (a,b) SVR and (c,d) DT for the “Golden Delicious” apples These diagrams compare the performance of algorithms in terms of RMSE variations and average correlation.
